# Depletion of mitochondrial reactive oxygen species downregulates epithelial-to-mesenchymal transition in cervical cancer cells

**DOI:** 10.18632/oncotarget.13612

**Published:** 2016-11-25

**Authors:** Galina Shagieva, Lidiya Domnina, Olga Makarevich, Boris Chernyak, Vladimir Skulachev, Vera Dugina

**Affiliations:** ^1^ Department of Mathematical Methods in Biology, Belozersky Research Institute of Physico-Chemical Biology, Lomonosov Moscow State University, Moscow, Russia; ^2^ Faculty of Basic Medicine, Lomonosov Moscow State University, Moscow, Russia; ^3^ Department of Bioenergetics, Belozersky Research Institute of Physico-Chemical Biology, Lomonosov Moscow State University, Moscow, Russia; ^4^ Faculty of Bioengineering and Bioinformatics, Lomonosov Moscow State University, Moscow, Russia

**Keywords:** epithelial-to-mesenchymal transition, mitochondrial reactive oxygen species, ERK1/2, cervical cancer cells, SkQ1

## Abstract

In the course of cancer progression, epithelial cells often acquire morphological and functional characteristics of mesenchymal cells, a process known as epithelial-to-mesenchymal transition (EMT). EMT provides epithelial cells with migratory, invasive, and stem cell capabilities. Reactive oxygen species produced by mitochondria (mtROS) could be of special importance for pro-tumorigenic signaling and EMT.

In our study, we used mitochondria-targeted antioxidant SkQ1 to lower the mtROS level and analyze their role in the regulation of the actin cytoskeleton, adhesion junctions, and signaling pathways critical for tumorigenesis of cervical carcinomas. A decrease in mtROS was found to induce formation of β-cytoplasmic actin stress fibers and circumferential rings in cervical cancer SiHa and Ca-Ski cells. It was accompanied by an upregulation of E-cadherin in SiHa cells and a downregulation of N-cadherin in Ca-Ski cells. In SiHa cells, an increase in E-cadherin expression was accompanied by a reduction of Snail, E-cadherin negative regulator. A stimulation of mtROS by epidermal growth factor (EGF) caused a Snail upregulation in SiHa cells that could be downregulated by SkQ1. SkQ1 caused a decrease in activation of extracellular-signal-regulated kinases 1 and 2 (ERK1/2) in SiHa and Ca-Ski. EGF produced an opposite effect. Incubation with SkQ1 suppressed EGF-induced p-ERK1/2 upregulation in SiHa, but not in Ca-Ski cells. Thus, we showed that scavenging of mtROS by SkQ1 initiated reversal of EMT and suppressed proliferation of cervical cancer cells.

## INTRODUCTION

Functioning of epithelial tissues requires stable intercellular contacts and cell polarity. Strict tissue organization is lost in epithelial tumors. Tumor epithelial cells usually discard basoapical polarity and reorganize cytoskeleton and adhesion junctions to acquire morphological and functional characteristics of mesenchymal cells. This process is known as EMT [1, 2]. EMT is a fundamental process involved in the regulation of embryonic development during morphogenesis, but it also plays a role in tumor progression and organ fibrosis [3–5]. EMT molecular changes include a downregulation of epithelial markers, such as E-cadherin, alpha-catenin and keratins, as well as a rise in expression of mesenchymal proteins N-cadherin and vimentin. EMT helps cells to become mobile. EMT endues cells with migratory, invasive, and stem cell capabilities [6, 7]. Escaping from the epithelial environment allows oncogene-expressing cells to proliferate and evolve [8].

The role of reactive oxygen species (ROS) and redox homeostasis in tumor progression is controversial. ROS are involved in the anticancer control mechanisms, apoptosis and cellular senescence. Most of the modern anticancer therapies rely on the ROS-induced killing of tumor cells, but this strategy results in strong side effects in normal tissues. On the other hand, the level of ROS is usually higher in malignant cells [9], and elevated ROS stimulate tumor cell proliferation, motility, and pro-survival signaling pathways contributing to tumorigenesis. Nevertheless, several clinical trials failed to show beneficial effects of dietary antioxidant supplements in primary cancer prevention and therapy [10]. This failure might be due to low efficiency of antioxidants in scavenging of the specific ROS responsible for tumorigenic signaling. Antioxidants can impede the intrinsic anticancer defense (apoptosis and cell senescence) as well as the effectivity of chemotherapy. Recent reports on the stimulation of melanoma metastases by antioxidants [11, 12] aroused wide public concern.

Targeting antioxidants to mitochondria is a promising approach since mtROS could be of special importance for pro-tumorigenic signaling [13]. Gene knockdown of MnSOD (mitochondrial superoxide dismutase isoform) resulted in mesenchymal-to-epithelial transition in aggressive breast cancer cell lines while induced MnSOD expression promoted EMT phenotype. The authors suggested that EMT was promoted by hydrogen peroxide produced by MnSOD in mitochondria [14]. An additional benefit of the mitochondria-targeted antioxidants based on lipophilic cations comes from a possible selective accumulation of these compounds in tumor cells. This phenomenon was described for the lipophilic cationic fluorescent probe Rhodamine 123 in 1982 [15], confirmed later for other cationic compounds, but still remains unexplained.

The first attempt to analyze the effect of mitochondria-targeted antioxidants on tumorigenesis was reported by our laboratory [16]. The efficient mitochondria-targeted antioxidant SkQ1 [17] suppressed a spontaneous tumor (predominantly lymphomas) development in homozygous *p53* knockout mice and inhibited the growth of human colon carcinoma HCT116/p53^−/−^ xenografts in athymic mice [16]. *In vitro* studies demonstrated that SkQ1 reversed the morphological transformation of Ras- and SV40-transformed p53^−/−^ fibroblasts and HCT116/p53^−/−^ cells [16]. A similar action (both *in vivo* and *in vitro*) of the antioxidant N-acetyl-L-cysteine (NAC) at more than 1,000,000 times higher doses indicated that the effects of SkQ1 were due to mtROS scavenging. Several mitochondria-targeted antioxidants designed as conjugates of triphenylphosphonium cation (TPP^+^) with nitroxides (Mito-CP, Mito-TEMPO) [18, 19] or a vitamin E analog (Mito-Vitamin-E) [21], also inhibited the tumor cell proliferation *in vitro* and the growth of tumor xenografts *in vivo*. Mito-CP acetamide (Mito-CP-Ac), an analog of Mito-CP containing TPP^+^ lacking the antioxidant activity, also inhibited proliferation of several tumor cell lines via an undiscovered mechanism [22]. A conjugate of TPP^+^ with ubiquinol (MitoQ) [23], the pioneering mitochondria-targeted antioxidant, inhibited proliferation of two breast cancer cell lines, but worked as a pro-oxidant, as revealed by the activation of Nrf2, a master regulator of the cellular response to oxidative stress [24].

In the present study we used SkQ1 to control mtROS and analyze their role in the regulation of the actin cytoskeleton, adhesion junctions and signaling pathways critical for the tumorigenesis of cervical carcinomas.

## RESULTS

### SkQ1 treatment changed actin cytoskeleton organization and proliferation in cervical cancer cells

The loss of epithelial phenotype by cells during EMT is connected with cytoskeleton rearrangement. Non-muscle β- and γ-actins are ubiquitously expressed in almost all human cells [24, 25]. Amount and distribution of two cytoplasmic actins – β-cytoplasmic actin (β-actin hereafter) and γ-cytoplasmic actin (γ-actin hereafter) are different in cancer and normal epithelial cells [27]. Cytoplasmic actins are organized in different cytoskeletal structures and connected to distinct cell junctions. β-Actin is predominant in stress fibers, circular bundles, contractile mitotic rings, and adhesion junctions (AJ). γ-Actin is connected to tight junctions [28] and is organized in cortical and lamellar networks [29].

**Figure 1:**
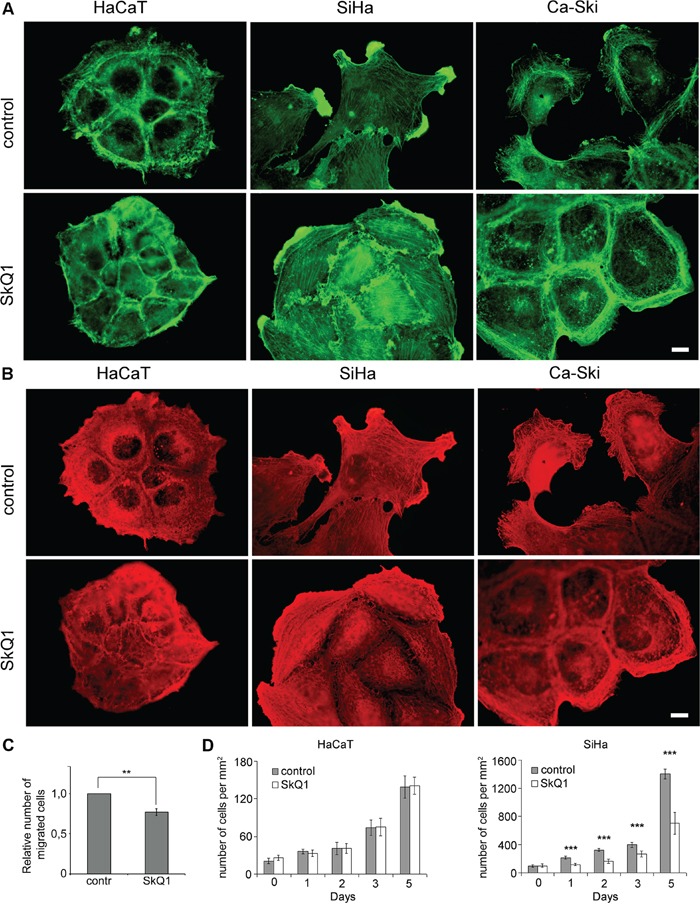
SkQ1 induced reorganization of cytoplasmic actins and inhibited proliferation of cervical cancer cells **A**. β-cytoplasmic actin in the control or cells treated with SkQ1 (40 nM for 3 days). **B**. γ-cytoplasmic actin in the control or cells treated with SkQ1 (40 nM for 3 days); immunofluorescence microscopy, scale bar 10μm. **C**. Transwell migration assay performed on SiHa treated with SkQ1 (40 nM), time point 16 hours. Statistically significant difference of data by Mann–Whitney test is marked (**) for p < 0.01. **D**. Influence of SkQ1 (40 nM) treatment on SiHa and HaCaT cells proliferation; phase contrast microscopy. Statistically significant difference of data by Student t-test is marked (***) for p < 0.001.

HaCaT are spontaneously transformed immortal keratinocytes from histologically normal adult human skin. HaCaT cells are polygonal in shape, form islands in culture and are similar to normal epithelial cells. Nevertheless HaCaT cells are aneuploid [30] and have mutations in *TP53*, tumor suppressor gene [31]. We used HaCaT as non-tumorigenic control line since (i) these cells were neither tumorigenic after subcutaneous injection nor invasive in a more sensitive transplantation assay [30], (ii) HaCaT transplants growing on glutaraldehyde-fixed collagen gels reconstituted an almost perfect epithelium with an increasing transition from ortho- to parakeratotic stratum corneum (containing nuclear remnants), which occurs normally in the epithelium of true mucous membrane of mouth and vagina [30], (iii) HaCaT cells were derived from the stratified squamous epithelium, just as cervical cancer cells did. In HaCaT cells β-actin was distributed diffusely in the cytoplasm, organized in circumferential rings at the peripheral cell area, and formed a network in the free lamellas. Incubation with SkQ1 (40 nM, 3 days) did not change general actin cytoskeleton organization but slightly influenced β-actin at the zone of free lamellas in HaCaT cells (Figure 1A).

We used two human cervical cancer cell lines- SiHa and Ca-Ski cells that represent different stages of a cancer progression. SiHa cells were obtained from a primary tumor while Ca-Ski cells were derived from a cervical carcinoma metastasis. Morphology of SiHa cells was heterogeneous; there were cells of both spindle-like and scattered types in the culture. Ca-Ski cells were mostly of scattered morphology. In both cancer cell lines β-actin was distributed diffusely in the cytoplasm and concentrated in lamellas and lamellipodia (Figure 1A). As a result of incubation with SkQ1, stress fibers and circumferential β-actin rings were formed. Dense γ-actin apical network did not change in HaCaT and SiHa cells after SkQ1 treatment. In Ca-Ski cells, SkQ1 stimulated a redistribution of γ-actin to the periphery (Figure 1B). Treatment with SkQ1 decreased SiHa cell motility in the Transwell assay (Figure 1C).

SkQ1 suppressed proliferation of SiHa cells, while proliferation of non-malignant HaCaT cells was not affected (Figure 1D). Antioxidant NAC (5mM) inhibited SiHa and HaCaT cells growth while structurally related to SkQ1 molecule without antioxidant capacity, C12TPP (40 nM), didn't influence proliferation of SiHa and HaCaT cells (data not shown). These data stimulated the further studies on the role of mtROS in EMT.

### SkQ1 improved adhesion junctions

Destabilization of intercellular contacts is one of the most important features of EMT. Actins are known to maintain stability of intercellular contacts. Both cytoplasmic actins were visualized at the apex of polarized epithelial cells in close proximity to intercellular contacts [28, 31, 32]. Adhesion junctions (AJ) are connected to β-actin [28] so the β-actin rearrangement induced by SkQ1 could influence AJ organization in cervical carcinoma cells. E-cadherin is a major AJ protein which is abundant in normal epithelial tissues, but is often suppressed in epithelial tumors [34]. We compared changes in location and amount of E-cadherin in non-neoplastic HaCaT and cervical cancer cells in response to mtROS depletion.

**Figure 2:**
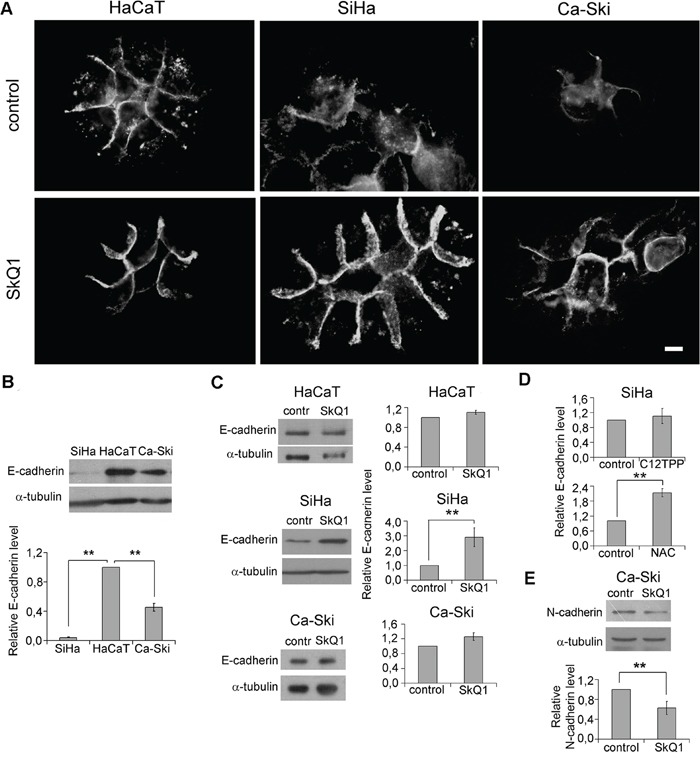
SkQ1 stimulated expression of E-cadherin and inhibited expression of N-cadherin in cervical cancer cells **A**. Distribution of E-cadherin in HaCaT, SiHa, and Ca-Ski cells in control cells and cells treated with SkQ1 (40 nM, 7 days); immunofluorescence microscopy, scale bar 10μm. **B**. E-cadherin expression in tumor SiHa and Ca-Ski cells compared to non-tumorigenic HaCaT cells; western blot analysis. **C**. E-cadherin expression in HaCaT, SiHa, and Ca-Ski cells in response to SkQ1 (40 nM, 7 days); western blot analysis. **D**. E-cadherin expression in SiHa cells treated with C12TPP (40 nM, 7days) or NAC (5mM, 7 days); densitometric analysis of western blots. **E**. SkQ1 (40 nM, 7 days) decreased N-cadherin content in Ca-Ski cells; western blot analysis. Statistically significant difference of data by Mann–Whitney test is marked (**) for p < 0.01.

Distribution and amount of E-cadherin in HaCaT cells was not affected by SkQ1 treatment (Figure 2A, 2C). E-cadherin amount in SiHa and Ca-Ski cells was significantly lower than in HaCaT cells (Figure 2B). E-cadherin was diffusely distributed in the cytoplasm with a higher concentration in the AJ zone of SiHa cells (Figure 2A). In Ca-Ski cells, E-cadherin was predominantly located in the AJ zone. SkQ1 stimulated E-cadherin expression in SiHa cells (Figure 2C) and induced a redistribution of E-cadherin to the AJ zone in SiHa and Ca-Ski cells (Figure 2A), where it was co-localized with β-actin (data not shown). C12TPP (40 nM, 7days), did not change E-cadherin expression and distribution in all three cultures (Figure 2D, for SiHa). Treatment with the ROS scavenger NAC (5mM, 7 days) increased E-cadherin level in SiHa cells (Figure 2D). Metastatic Ca-Ski cells express both epithelial E-cadherin and mesenchymal N-cadherin, a marker of advanced EMT. We observed N-cadherin downregulation caused by SkQ1 in Ca-Ski cells (Figure 2E). These data indicated that expression and distribution of E-cadherin as well as expression of N-cadherin depend on mtROS in cervical carcinoma cells.

### SkQ1 downregulated transcription factor Snail

**Figure 3:**
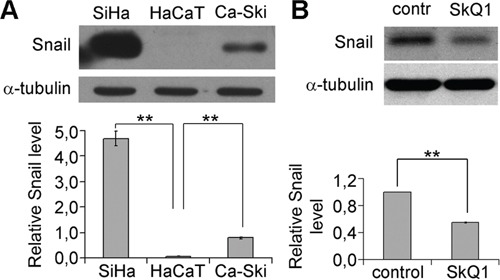
SkQ1 decreased Snail content in SiHa cells **A**. Snail expression in HaCaT, SiHa and Ca-Ski cells; western blot analysis. **B**. Snail expression in SiHa cells in response to SkQ1 (40 nM, 1 day); western blot analysis. Statistically significant difference of data by Mann–Whitney test is marked (**) for p < 0.01.

Expression of E-cadherin can be regulated by different mechanisms [35]. Transcriptional repression is a fundamental mechanism for the dynamic silencing of E-cadherin gene. E-cadherin expression is downregulated by various transcription factors including Snail, Slug, Twist [36]. The amount of Snail in SiHa cells was significantly higher than in HaCaT while in Ca-Ski cells it was intermediate (Figure 3A). We observed a reduction of Snail in SiHa cells as a result of incubation with 40 nM SkQ1 (Figure 3B); effect was detected after one day of treatment and lasted until the 7th day. Snail level in Ca-Ski cells did not change after SkQ1 treatment (data not shown). These data indicated that mtROS inhibited E-cadherin expression via stimulation of Snail in SiHa cells while the other mechanisms were responsible for E-cadherin suppression in Ca-Ski cells.

### SkQ1 inhibited ERK1/2 activity and upregulated dual-specificity protein phosphatase DUSP6

**Figure 4:**
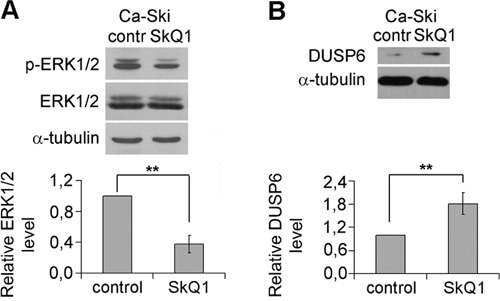
Inhibition of ERK1/2 phosphorylation (activation) and upregulation of DUSP6 expression by SkQ1 in Ca-Ski cells **A**. ERK1/2 phosphorylation altered as a result of SkQ1 (40 nM, 6 days) treatment; **B**. SkQ1 (40 nM, 6 days) influenced DUSP6 expression; western blot analysis. Statistically significant difference of data by Mann–Whitney test is marked (**) for p < 0.01.

Transcription factor Snail could be activated by ERK1/2 pathway [37–39]. ERK-1 and ERK-2 (ERK1/2 hereafter) belong to mitogen-activated protein kinases (MAPK) that control proliferation, motility, adhesion, invasion, survival and play an important role in tumor progression. Thus, we investigated the possible involvement of MAPK signaling pathways in SkQ1-induced changes in cervical cancer cells. We identified that SkQ1 treatment decreased ERK1/2 activation in SiHa and Ca-Ski cells (Figure 4A); the effect was detected after 45 min of incubation and lasted until the 7th day. No significant changes in phosphorylation of another MAPK, p38 were observed after incubation with SkQ1 (data not shown).

Dual-specificity protein phosphatases (DUSPs) dephosporylate phosphothreonine and phosphotyrosine residues and inactivate MAPK. DUSP6 is a cytoplasmic enzyme which selectively dephosphorylates ERK1/2 [40]. We observed the increase in DUSP6 content in Ca-Ski cells as a result of SkQ1 treatment (Figure 4B), that could be responsible for ERK1/2 inhibition. DUSPs and MAPKs form feedback loops, each controlling activities of the other. Active ERK1/2 phosphorylate transcription factors ETS1/2 to downregulate activity of DUSP6 promoter and inhibit its expression [41]. Moreover ERK1/2 can phosphorylate DUSP6 and promote its subsequent degradation by the proteasome [42]. Thus ERK1/2 exert a positive feedback loop on their own activity by downregulation of DUSP6. Inhibition of ERK1/2 by mtROS depletion probably occurs, at least in part, due to interruption of these feedback mechanisms. The direct stimulation of DUSP6 activity by SkQ1 is also possible since this enzyme has cysteine in active site and could be inactivated by ROS.

### EGF-induced signaling leading to EMT progression was inhibited by SkQ1

**Figure 5:**
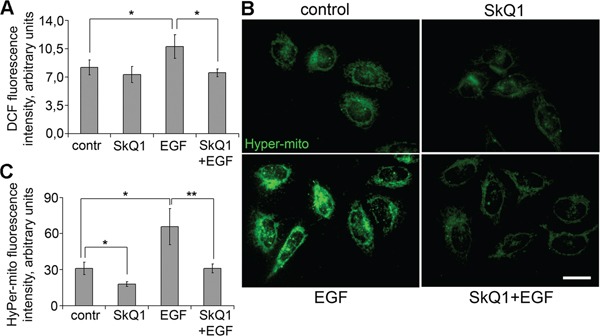
SkQ1 prevented accumulation of cytoplasmic and mitochondrial ROS induced by EGF in SiHa cells **A**. Effect of SkQ1 (40 nM, 7 days) on accumulation of cytoplasmic ROS with or without and EGF (10 ng/ml, 10 min); DCFH-DA assay, flow cytometry. **B**. and **C**. SkQ1 (20 nM, 1 day) influenced accumulation of mtROS with or without EGF (10 ng/ml, 2 hours); cells with mito-Hyper sensor of H_2_O_2_, fluorescence microscopy, scale bar 20μm. Statistically significant difference of data by Student's t-test is marked (*) for p < 0.05 and (**) for p < 0.01.

**Figure 6:**
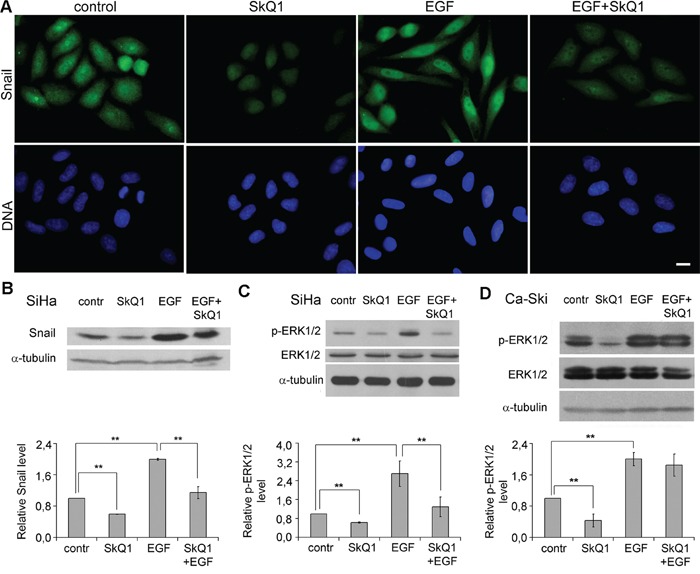
SkQ1 suppressed EGF induced signaling in cervical cancer cells **A**. and **B**. SkQ1 (20 nM, 7 days) altered Snail expression induced by EGF (5 ng/ml, 3 days) in SiHa cells; immunofluorescence microscopy (A), scale bar 10μm; western blot analysis (B). **C**. SkQ1 (20 nM, 7 days) inhibited ERK1/2 activation induced by EGF (5 ng/ml, 3 days) in SiHa cells; western blot analysis. **D**. Effect of SkQ1 (20 nM, 6 days) on ERK1/2 activation induced by EGF (5 ng/ml, 1 day) in Ca-Ski cells; western blot analysis. Statistically significant difference of data by Mann–Whitney test is marked (**) for p < 0.01.

EGFR is known to activate EMT-inducing signaling pathways in cervical cancer [43]. EGFR signaling depends on H_2_O_2_ generation [44] and activation of ERK1/2 [45]. We observed simulation of EMT phenotype via EGF in SiHa and Ca-Ski cells and investigated the effect of SkQ1 on the process. Treatment with EGF induced accumulation of Snail in nucleus and significantly increased overall amount of Snail in SiHa cells (Figure 5A, 5B). Incubation with EGF stimulated ERK1/2 phosphorylation in SiHa and Ca-Ski cells (Figure 5C, 5D). Pre-incubation with SkQ1 downregulated Snail (Figure 5B) and ERK1/2 phosphorylation (Figure 5C) in SiHa cells, but did not affect ERK1/2 activation caused by EGF in Ca-Ski cells (Figure 6D). These data indicate that SkQ1 induced reverse of epithelial-to-mesenchymal transition in cervical cancer cells at least in part, due to inhibition of EGFR signaling.

### SkQ1 decreased accumulation of hydrogen peroxide in mitochondria

SkQ1 is a mitochondria-targeted antioxidant [16, 17], but the direct measurements of SkQ1 effects on ROS accumulation in mitochondria are rarely performed. We measured intracellular ROS using dichlorodihydrofluorescein diacetate (DCFH-DA) and flow cytometry analysis. SkQ1 did not change significantly basic intracellular ROS, but it downregulated EGF-induced ROS increase in SiHa cells (Figure 6A). For measurements of ROS accumulation in mitochondria we used the lentiviral construct expressing mitochondria-targeted recombinant fluorescent protein HyPer-mito, a sensor for hydrogen peroxide in mitochondria [46]. We observed that hydrogen peroxide was reduced after SkQ1 treatment and increased after incubation with EGF in SiHa cells. SkQ1 suppressed the EGF-induced hydrogen peroxide accumulation in mitochondria of SiHa cells (Figure 6B, 6C). These data indicated that inhibition of ROS accumulation in mitochondria as a result of SkQ1 treatment could be responsible for its action on cervical cancer cells.

## DISCUSSION

In agreement with earlier findings [16], we demonstrated that the mitochondria-targeted antioxidant SkQ1 (40 nM) inhibited proliferation of cervical carcinoma SiHa cells while proliferation of non-tumorigenic keratinocytes HaCaT was not affected. Lipophilic cation C12TPP (which resembles SkQ1 but does not contain antioxidant plastoquinolyl residue) did not influence proliferation of SiHa cells. Compared with SkQ1, non-targeted antioxidant NAC (5mM) inhibited proliferation of SiHa cells at a significantly higher concentration, indicating that scavenging of mtROS was critical for the antiproliferative action of SkQ1. Effective inhibition of the ROS accumulation in mitochondria of SiHa cells by SkQ1 was shown using a mitochondria-targeted hydrogen peroxide sensor HyPer-mito.

The antiproliferative action of SkQ1 could be mediated by reorganization of β-actin cytoskeleton and inhibition of MAPK signaling in cervical cancer cells. In addition to architectonics, intracellular transport and motility of the cells, actin cytoskeleton is important for proliferation and intracellular signaling. Decreased β-actin coupled with abundant γ-actin was detected in cervical and breast tumors compared to corresponding normal tissues [26, 32]. Previously [47] we showed that β-actin acts as a tumor suppressor, inhibiting cell proliferation and invasion *in vitro* and tumor growth *in vivo*. ERK1/2 activation is linked to β-actin decrease and γ-actin upregulation in carcinoma cells. Contraction and adhesion are linked to β-actin, whereas γ-actin is predominantly organized in a cortical network which is necessary for the maintaining cell architecture and motility of normal fibroblasts and epithelial cells [29]. The loss of actin bundles correlates with an increase of migratory activity and metastatic potential of tumor cells. We conclude that a reorganization of β-actin into circular bundles as a result of SkQ1 treatment contributed to suppression of migration and proliferation in cervical cancer cells. The precise mechanisms by which mtROS regulate β-actin cytoskeleton reorganization require further investigation.

Cytoplasmic β-actin interacts with E-cadherin, principle AJ protein [28]. Rearrangement of β-actin and recruitment of β-actin bundles to AJ (probably due to E-cadherin upregulation) improved AJ in cervical carcinoma cells treated with SkQ1. Decreased expression of E-cadherin is thought to be the prototypical marker of EMT [48]. The loss of E-cadherin is associated with a tumor progression in various epithelial cancers while recovery of E-cadherin adhesion complex suppresses invasiveness of different tumor cells [48, 49].

SkQ1 stimulated E-cadherin expression in SiHa cells and induced a redistribution of E-cadherin to the AJ zone in the both SiHa and Ca-Ski cells. Increased expression of E-cadherin in SiHa was accompanied by downregulation of Snail. Snail is a transcriptional repressor of E-cadherin which induces tumor invasion during cancer progression and also triggers EMT during the embryonic development of diverse species from Drosophila to mammals. No deviations occurred during the embryogenesis in mice receiving SkQ1 [M.V. Skulachev, personal communication].

The expression of Snail is stimulated by ERK1/2 [37–39] and positive feedback regulation of ERK2 by Snail was observed in breast cancer cells MCF-7 [51]. SkQ1 decreased ERK1/2 activation in cervical cancer cells suggesting that inhibition of Snail expression by SkQ1 could be mediated via ERK1/2 inhibition. Snail acts downstream of EMT-inducing signaling pathways which can be activated by different growth factors including EGF. Epidermal growth factor receptor (EGFR) is overexpressed in 70% to 90% of analyzed cervical cancer cases [52]. Immunofluorescent study of surgical specimens indicated that cervical carcinoma progression was accompanied by EGFR overexpression and decreased E-cadherin content that correlated with Snail upregulation [43]. To study the role of EGFR signaling in cervical cancer cells we stimulated EMT progression with exogenous EGF. It was shown that activation of ERK1/2 and Snail by EGF in SiHa cells was prevented by SkQ1 suggesting that EGFR signaling critically depends on mtROS.

EGF increased accumulation of mitochondrial ROS as well as cytoplasmic ROS and SkQ1 prevented the both effects indicating that mtROS could be the primary target of EGFR signaling. One of the possible mechanisms of EGFR-dependent mtROS production is related to activation of p66shc protein that can generate ROS in mitochondria [53]. EGFR pathway is controlled by redox-dependent mechanisms at various levels. It was shown that p66shc-dependent mtROS stimulated EGFR and platelet-derived growth factor receptor signaling via oxidation and inhibition of protein tyrosine phosphatases [54]. SkQ1 inhibited mtROS in the cells overexpressing p66shc [55] so downregulation of EGFR signaling in cervical cancer cells was probably mediated by inhibition of p66shc-dependent mtROS production.

Oxidative inhibition of protein tyrosine phosphatases is the most general mechanism regulating signal transduction. MAPK phosphatases (MKPs) are dual-specificity phosphatases (DUSPs) that are highly specific for the MAPKs and can be regulated at the level of gene expression. This subclass of protein phosphatases is capable of direct binding to MAPKs, resulting in dephosphorylation of phosphothreonine and phosphotyrosine residues and inactivation of MAPKs. DUSP6 (MKP-3) is a cytoplasmic enzyme which selectively dephosphorylates and inactivates ERK1/2 [56]. In ovarian cancer cells oxidative stress resulted in DUSP6 ubiquitination and proteasomal degradation followed by ERK1/2 activation, tumor progression and development of chemoresistance *in vitro* and *in vivo* [57]. ROS scavenging by an antioxidant N-acetyl-L-cysteine increased DUSP6 expression as well as dephosphorylation of ERK1/2, and inhibited ovarian cancer cells proliferation [57]. Increased ROS production also resulted in the antioxidant response element (ARE)/Nrf2-dependent upregulation of the transcription factor ETS1 [58]. Notably ERK1/2 can phosphorylate transcription factors ETS1/2 and inhibit DUSP6 expression [41]. At the same time, ERK1/2 directly phosphorylate serines 159 and 197 of DUSP6 and stimulated its proteasomal degradation [42]. These data demonstrated that there are several pathways for ROS-dependent dowregulation of DUSP6. Since SkQ1 stimulated DUSP6 and prevented ERK1/2 activation in Ca-Ski cells the key role of mtROS in these pathways could be suggested.

We demonstrated that scavenging of mtROS with SkQ1 resulted in actin cytoskeleton reorganization and ERK1/2 inactivation in both SiHa and Ca-Ski cells, but downregulation of Snail followed by increase in E-cadherin expression was detected in SiHa cells only. SiHa and Ca-Ski cells display two different stages of cancer progression as they were derived from primary tumor and cervical carcinoma metastasis, respectively. ERK1/2-dependent Snail activation at the early stages of tumorigenesis leads to rapid and effective repression of E-cadherin that promotes EMT to initiate invasion. This pathway critically depends on increased mtROS production as we saw in SiHa. Maintenance of the motile phenotype in invading tumor cells depends on weaker but more widely expressed repressors Slug, E47, and SIP1 while Twist1 plays a key role in distant metastasis [59]. In Ca-Ski cells derived from metastasis E-cadherin is partially replaced by mesenchymal N-cadherin that is known to form the weaker intercellular adhesions [2]. Moreover, N-cadherin contributed to sustained activation of the MAPK-ERK pathway, leading to transcription of matrix metalloprotease MMP-9 gene and cellular invasion [60]. Forced expression of N-cadherin in well-differentiated breast cells increases invasiveness of cells even in presence of high E-cadherin expression [61]. SkQ1 decreased expression of N-cadherin in Ca-Ski cells indicating that mtROS contributed to EMT promotion in the cells derived from metastasis of cervical carcinoma. In Ca-Ski cells EGF-induced ERK1/2 activation was not affected by SkQ1 in contrast to SiHa cells. This difference occurs at least in part because EGFR expression in Ca-Ski is about 6 times higher than in SiHa cells [62].

Tumor-initiating cells (TICs) from carcinomas of several different types carry distinct mesenchymal features, that suggests they have passed through the EMT which helped them to acquire properties of stem cells [63]. TICs are important targets for cancer therapy owing to their higher tumor-initiating ability and elevated resistance to chemotherapy [64]. Upregulation of E-cadherin expression diminishes the number of TICs and decelerates tumor growth in human A549 lung adenocarcinoma cells [65]. EMT reversal in mesenchymal derivatives of human mammary epithelial cells stimulated them to enter epithelial non-stem-like state that made chemotherapy more cytotoxic to them [66].

In conclusion, we showed that scavenging of mtROS by SkQ1 initiated reversal of EMT in cervical carcinoma cells as revealed by an upregulation of epithelial markers and a downregulation of mesenchymal markers. These findings suggest that mitochondria-targeted antioxidants could be considered as potential partner drugs in a combinational therapy of cervical cancers.

## MATERIALS AND METHODS

### Cell culture and chemicals

SiHa and Ca-Ski cells were obtained from the American type culture collection (ATCC): SiHa cell line (ATCC #HTB-35) was derived from a surgical material of cervical carcinoma; cells contain one or two copies of the human papilloma virus 16 type (HPV 16) DNA integrated in the chromosome 13. Ca-Ski cell line (ATCC #CRL-1550) was derived from a surgical specimen of cervical carcinoma metastasis into the intestinal mesentery; cells contain integrated DNA of HPV 16 (about 600 copies per cell) and fragments of HPV 18 DNA. HaCaT are spontaneously immortalized *in vitro* keratinocytes from a surgical specimen of histologically normal human skin [30].

Cell lines were maintained in Dulbecco's modified Eagle media (DMEM) with 5% fetal bovine serum (HyClone), 5мМ glutamic acid (PanEco) at 37°C in the atmosphere of air containing 5% CO2.

SkQ1 (10-(6’-plastoquinonyl) decyltriphenylphosphonium) is a conjugate of TPP^+^ with the plant electron carrier plastoquinone [17]. C12TPP is a structurally related to SkQ1 molecule lacking the plastoquinol moiety. Both SkQ1 and C12TPP in their cationic forms can penetrate the planar bilayer phospholipid membrane and accumulate in isolated mitochondria or in mitochondria in human cells in culture. Optimal according to our observations concentrations 20-40 nM were used for SkQ1 and C12TPP. For EMT stimulation via human epidermal growth factor (EGF, Cell Signaling) cells were maintained in DMEM with 1% fetal bovine serum and 5мМ glutamic acid. For short-term experiments (5 min - 2 hours) we used 10 ng/ml of EGF, for long-term experiments we used 5 ng/ml of EGF. NAC (Sigma) was used in concentration 5mM.

### Immunofluorescence microscopy

The cells were cultivated on the glass cover slips washed with DMEM containing 20 mM 4-(2-hydroxyethyl)-1-piperazineethanesulfonic acid (HEPES) at 37°C. The cells were fixed for 15 min with 1% paraformaldehyde on a serum-free DMEM (with 20 mM HEPES), and were extracted using cold methanol for 5 min at −20°C for subsequent antibody staining. The following antibodies were used: mouse monoclonal antibodies against cytoplasmic β-actin (4C2 clone), cytoplasmic γ-actin (2A3 clone; Dugina et al., 2009), E-cadherin (BD Transduction Laboratories), rat antibodies against Snail (Cell Signaling). The secondary antibodies were goat fluorescein- and rhodamine-conjugated antibodies against mouse immunoglobulins IgG2a, IgG2b, and IgG1 (Southern Biotechnology). Nuclear staining was performed using DAPI (Sigma). Immunofluorescence was observed using Axioplan microscope with 40×/0.75 and 100×/1.3 Plan-Neofluar lenses (Carl Zeiss).

### Western blot analysis

Cells were extracted with cold sample buffer (62.5 mM Tris-HCl, pH 6.8, 2% sodium dodecyl sulfate (SDS), 10% glycerin, 50 mM dithiothreitol, 0.01% bromophenol blue). Lysates were separated in 10% SDS polyacrylamide gel and transferred onto polyvinylidene fluoride membrane (Amersham GE Healthcare). After blocking the nonspecific binding using bovine serum albumin, the membranes were incubated with specific antibodies against β-actin, γ-actin, E-cadherin, Snail; rabbit antibodies against ERK1/2(p42/44) and phosphorylated ERK1/2 (p-p42/44) (Cell Signaling), p38 and p-p38 (Cell Signaling), DUSP6 (Cell Signaling). We used α-tubulin as a loading control. The membranes were incubated with secondary antibodies bound with horseradish peroxidase (Amersham GE Healthcare). The membranes were developed using the chemiluminescence technique with ECL reagents (Amersham GE Healthcare) according to producer's protocol. The resulting films were scanned and analyzed densitometrically with ImageJ 1,37C software (NIH, http://rsb.info.nih.gov/ij/).

### Detection of reactive oxygen species

For the intracellular ROS measurement, we incubated SiHa cells with the fluorescent dye 2′,7′-dichlorodihydrofluorescein diacetate, DCFH-DA («Invitrogen», 1.8 μM) for 10 min on a serum-free DMEM medium at 37°C in the dark. After the oxidation by ROS dichlorodihydrofluorescein transforms to the highly fluorescent dichlorofluorescin (DCF). Then cells were detached with the trypsin-EDTA solution and ROS accumulation was measured by flow cytometry with Beckman Coulter Cytomics FC500 at the 525 nm wavelength, with excitation at the 488 nm wavelength.

For measurements of ROS accumulation in mitochondria we used the lentiviral construct expressing mitochondria-targeted recombinant fluorescent protein HyPer-mito, a sensor for hydrogen peroxide in mitochondria [46] introduced in SiHa cells. Cells were cultivated on the glass cover slips and washed with DMEM containing 20 mM HEPES at 37°C. Cells were fixed for 15 min with 1% paraformaldehyde on a serum-free DMEM (with 20 mM HEPES). HyPer-mito fluorescence was observed using Axioplan microscope with 100×/1.3 Plan-Neofluar lenses (Carl Zeiss) and was analyzed with ImageJ 1,37C software.

### Transwell migration assay

The assay was performed using transwell chambers with 8-μm pore-size membranes (Corning Life Sciences) according to manufacturer protocol with 10^4^ SiHa cells from 0,1 to 5% of FBS. The migration activity was quantified by the number of migrated cells (time point - 16 hours) from 10 fields per chamber in 3 independent experiments.

### Statistical analysis

Results are presented indicating mean ± standard error of the mean of at least three independent experiments. Intergroup differences were analyzed by the Mann–Whitney U test or Student's t-test when applicable. Values of p < 0.001 (***), p < 0.01 (**), and p < 0.05 (*) were considered as statistically significant.

## Abbreviations

(EMT): epithelial-to-mesenchymal transition
(ROS): reactive oxygen species
(mtROS): mitochondrial reactive oxygen species
(NAC): N-acetyl-L-cysteine
(ATCC): american type culture collection
(HPV): human papillomavirus
(DMEM): Dulbecco's modified Eagle media
(EGF): epidermal growth factor
(SDS): sodium dodecyl sulfate
(HEPES): 4-(2-hydroxyethyl)-1-piperazineethanesulfonic acid
(AJ): adhesion junctions
(MAPK): mitogen-activated protein kinases
(DUSPs): extracellular-signal-regulated kinases 1 and 2 (ERK1/2), dual-specificity protein phosphatases
(EGFR): epidermal growth factor receptor
(TNF): tumor necrosis factor
(MET): mesenchymal-to-epithelial transition
(TICs): tumor-initiating cells
